# Self-Management Support Apps for Spinal Cord Injury: Results of a Systematic Search in App Stores and Mobile App Rating Scale Evaluation

**DOI:** 10.2196/53677

**Published:** 2024-12-19

**Authors:** Renaldo M Bernard, Vanessa Seijas, Micheal Davis, Anel Volkova, Nicola Diviani, Janina Lüscher, Carla Sabariego

**Affiliations:** 1 Swiss Paraplegic Research Nottwil Switzerland; 2 Faculty of Health Sciences and Medicine University of Lucerne Lucerne Switzerland; 3 Center for Rehabilitation in Global Health Systems University of Lucerne Lucerne Switzerland

**Keywords:** mobile phone, mobile health, mHealth, eHealth, telemedicine, telehealth, spinal cord injury, self-management, internet-based intervention, world wide web, systematic review, review

## Abstract

**Background:**

The use of mobile technology to meet health needs, widely referred to as mobile health (mHealth), has played a critical role in providing self-management support for chronic health conditions. However, despite its potential benefits, mHealth technologies such as self-management support apps for spinal cord injury (SCI) have received little research attention, and an understanding of their public availability is lacking. Therefore, an overview of these apps is needed to complement findings from the literature for a complete understanding of mHealth self-management support tools for SCI to support the selection and improvement of existing apps and the development of new ones.

**Objective:**

This study aimed to identify and describe quantity, quality, focus, strengths, and weaknesses of self-management support apps for SCI available on major mobile app digital distribution platforms.

**Methods:**

A systematic search of the Google Play Store and Apple App Store was conducted to identify and summarize apps for SCI that have been updated since 2017. A supplementary systematic literature review was conducted across 11 bibliographic databases to identify publications that provided more detailed descriptions of the identified apps than what is typically available in app stores. The data synthesis was guided by self-management tasks and skills taxonomies. The PRISMA (Preferred Reporting Items for Systematic Reviews and Meta-Analyses) guidelines informed the reporting.

**Results:**

The 13 apps included in the final synthesis were launched between 2013 and 2021, mostly originating in the United States, with availability in 72 countries and support for 14 languages. Most apps used the Android operating system (10/13, 77%), while 31% (4/13) used iOS. The identified apps mainly focused on activities of daily living, physical activity promotion, health literacy, and therapeutic exercise. All 3 self-management tasks (medical, role, and emotional management) and most self-management skills and support activities were supported by the apps. The mean Mobile App Rating Scale score was 3.86 (SD 0.54), indicating good overall quality. No publications were found describing these apps.

**Conclusions:**

Despite their good overall quality, as measured by the Mobile App Rating Scale assessment, the 13 identified apps, alone or combined, do not appear to offer a comprehensive self-management approach that incorporates theory-based strategies. Besides working to improve comprehensiveness, future research and practice should consider adopting new technologies, such as artificial intelligence, to enhance future self-management support apps for SCI. Furthermore, adopting new app development methods, such as low-code development platforms, could help reduce barriers to development, such as time, cost, and securing scarce expertise.

## Introduction

### Background

Spinal cord injury (SCI) is a life-altering condition caused by traumatic or nontraumatic events, leading to temporary or permanent disruption of the normal motor, sensory, or autonomic functions of the spinal cord. It affects the physical, social, and psychological well-being of the patient and places an important burden on health care systems, families, and communities [1]. SCI is a chronic health condition that causes multiple limitations in daily functioning, such as limitations in walking, eating, grooming, working, or caring for oneself, and secondary health conditions, such as spasticity, constipation, urinary tract infections, chronic pain, sexual dysfunction, fatigue, and mental health disorders [2,3]. Therefore, people with SCI frequently rely on specialized and costly health care services, often facing significant unmet health care needs and challenges, particularly in low- and middle-income countries. These challenges are primarily due to the costs of health care services, transportation, and limited service availability [4]. Self-management support, such as the provision of social support and equipment [5], is essential for people with SCI to independently manage the challenging symptoms, treatment, and lifestyle changes associated with having a SCI [6-8].

Besides traditional self-management support, such as in-person counseling sessions, the adoption of mobile health (mHealth) self-management support is growing in the self-management of chronic health conditions such as SCI [9]. mHealth involves using mobile and wireless information and communication technologies, including smartphones, tablets, and wearables, to support meeting health needs [10]. Therefore, mHealth provides more person-centered, available, accessible, and scalable self-management support options than many traditional alternatives, such as institutional- and paper-based options [11]. Smartphones have one of the highest adoption rates among mHealth technology [12,13] and are essential tools for improving diagnostics, personal health monitoring and tracking, access to health care, and the development of health literacy [14].

However, mHealth self-management support tools for SCI have seemingly received little research attention despite the unique self-management support necessitated by the distinctive pathophysiology of SCI. To the best of our knowledge, the most relevant overviews of these tools are provided by systematic literature reviews by Wellbeloved-Stone et al [15] and Bernard et al [16]. Although insightful, the first study does not account for the expected rapid increase in the development of mHealth tools over the last 6 years and identifies 1 tool [15], and both studies are biased toward published literature as they potentially neglect apps that are available through digital distribution platforms and are not mentioned in published literature [15,16]. While mHealth self-management support apps for chronic health conditions aim to assist people in honing self-management skills, there exists a need for apps targeting SCI due to its distinct pathophysiology [17]. For example, self-management for bladder impairments mainly involves managing incontinence in prostate cancer, Parkinson disease, and stroke, compared to bladder voiding dysfunction, urinary tract infections, urinary stones, and renal impairment in SCI [18].

Therefore, identifying available self-management support apps for SCI is important for achieving a more complete overview of mHealth self-management support tools for SCI that complements related findings from the literature. This overview can also be used as a basis for the development of a convenient reference collection of self-management support apps for SCI to support the selection and improvement of existing apps and the development of new ones.

### Objectives

The objective of this study was to identify and describe self-management support apps for SCI available on mobile app digital distribution platforms. This study aimed to (1) describe these apps in relation to their quantity and quality, coverage of self-management support activities and skills, and self-management areas targeted by self-management approaches; and (2) highlight associated strengths and weaknesses.

## Methods

### Overview

The study was conducted in 2 phases. In phase 1, a systematic search of app stores was conducted to identify and summarize self-management support apps for SCI. In phase 2, a systematic literature review was conducted to complement the work done in phase 1. This review aimed to identify publications that focused on designing, describing, evaluating, piloting, implementing, or improving apps previously identified in phase 1. The PRISMA (Preferred Reporting Items for Systematic Reviews and Meta-Analyses) guidelines [19] and their extension for literature searches [20] were used to guide reporting in both phases. There were deviations from the study protocol [21] due to unforeseen resource constraints. The study’s aims were narrowed in scope. When available in both stores, apps were reviewed based on device availability rather than prioritizing apps from a specific store. The IMS Institute for Healthcare Informatics app functionality scale [22] and the behavior change technique taxonomy (version 1) [23] were not used for app evaluation and synthesis, respectively.

### Search Strategy

#### Phase 1

Similar to recent systematic searches of app stores [24-26], the Google Play Store and Apple App Store were systematically searched via an application programming interface provided by 42Matters [27] using keywords for SCI to find eligible apps across regional marketplaces (Multimedia Appendix 1). These digital distribution platforms host the largest number of publicly available mobile apps globally for smartphones, tablets, and wearables [28]. Given the limited documentation for the application programming interface of 42Matters, researchers conducted preliminary searches and communicated with company representatives to enhance their understanding. To avoid missing relevant apps, the search was comprehensive, covering app titles, descriptions, and developer names. Additional apps were identified by looking at other apps by the same developers and through the “similar apps” and “you might also like” features on both app stores.

#### Phase 2

MEDLINE, CINAHL Complete, PsycINFO, IEEE Xplore Digital Library, ACM Digital Library, Embase, Scopus, Web of Science Core Collection, Academic Search Premier, LISTA, and Business Source Premier were searched to find eligible literature (Multimedia Appendix 1). The search terms used identified app titles and developer names from phase 1. The reference lists of the included articles were planned to be hand searched.

### Eligibility Criteria

#### Phase 1

To be eligible for inclusion, apps should target individual users with SCI for use primarily outside a clinical setting; be supportive of self-management in SCI; be available to all users within the corresponding regional marketplace; be accessible via a smartphone, tablet, or wearable (eg, smartwatch); be available in English; and be last updated after July 2017 and available up to July 2022. The fully featured version of the app was preferred over the limited version when priced at ≤US $10. In addition, the smartphone version was chosen over the tablet version, and the tablet was favored over the wearable version. Apps were excluded if a technical malfunction prevented access or use after 2 attempts. These criteria were selected as practical choices based on available resources to ensure the inclusion of apps that are relevant to individuals with SCI, accessible to a wide user base, up-to-date, and user-friendly across various device types, all while considering cost and technical functionality.

#### Phase 2

Publications were eligible for inclusion if they described an identified app from phase 1. Publications available in English were considered. Primary research studies, books, and gray literature (eg, conference proceedings, company websites, and professional publications) were also considered. Broad eligibility criteria were used to maximize the possibility of finding additional information to describe apps identified in phase 1.

### Eligibility Assessment

Three researchers (AV, MD, and RMB), having expertise in health science, psychology, and health technology, were involved in the screening process. They attended training sessions to ensure consistency in screening during phases 1 and 2, using a web-based spreadsheet and the web-based service Rayyan [29], without using its artificial intelligence features. Screeners completed a training set of 20% of assigned apps and publications. For phase 1, screeners (AV and RMB) were randomly assigned a screening set of app titles, descriptions, and screenshots, as well as of apps to download and screen their content. For phase 2, screeners (MD and RMB) were randomly assigned a screening set of publication titles, abstracts or summaries, and full texts. A second screening of at least 20% was conducted as part of the training set for apps and publications. Screening was independently conducted to reduce the risk of reviewer bias [30]. Conflicting screening decisions (ie, *include*, *maybe,* or *exclude*) were resolved collaboratively.

### Quality Assessment

Two reviewers (AV and MD), both health scientists, independently assessed the identified apps using the 23-item Mobile App Rating Scale (MARS) [31]. The MARS is a widely adopted and reliable tool for classifying and assessing mHealth app quality using 5 subscales: engagement (5 items), functionality (4 items), aesthetics (3 items), information quality (7 items), and a subjective app quality score (4 items) [32]. The review excluded the subjective quality dimension to ensure objectivity in the quality assessment process. Inconsistent ratings were resolved collaboratively. Interrater reliability was calculated for total and dimensional scores using the intraclass correlation coefficient [33]. A quality assessment was not planned for publications, as phase 2 aimed to provide supplementary descriptive information and not quality judgments.

### Data Extraction and Synthesis

The same researchers who were involved in the screening process completed data extraction. These researchers attended a training session to help ensure consistency and reliability in data extraction using a web-based data extraction form. The form was discussed and modified for increased clarity. One researcher extracted data from the identified apps, corresponding app store pages, and developer websites, and then, another researcher reviewed and verified the extracted data. The extracted data were collated and summarized by AV and RMB. Any discrepancies in data extraction among researchers were resolved collaboratively.

A widely accepted framework or clinical guideline regarding the key components of a SCI self-management intervention could not be found. Therefore, data extraction and synthesis were guided by the self-management approaches for people with chronic conditions by Barlow et al [6], the self-management skills taxonomy by Lorig and Holman [34] (detailed in Textbox 1), and the Practical Reviews in Self-Management Support taxonomy of self-management support activities [5] to help organize its findings.

**Self-management task and skill frameworks.**

**Self-management tasks [6]**

- Medical management: making health-related appointments, following treatment plans, tracking symptoms, and taking medication as directed
- Role management: organizing and coordinating the various everyday roles and responsibilities related to work, family, community, and self-care and adapting these roles as needed
- Emotional management: regulating and coping with emotions resulting from living with a condition in a healthy and effective manner

**Self-management skills [34]**

- Problem-solving: identifying problems and finding, implementing, and evaluating solutions
- Decision-making: weighing options and choosing the best course of action in response to changes in their condition
- Resource use: finding and effectively using resources
- Forming patient-provider partnerships: learning from and partnering with health care professionals to understand the patterns experienced with a condition, make informed decisions, and discuss related issues
- Action planning: developing a realistic action plan that can be confidently used to achieve a set goal
- Self-tailoring: developing and implementing personalized self-management strategies as needed

## Results

### Overview

**Figure 1:**
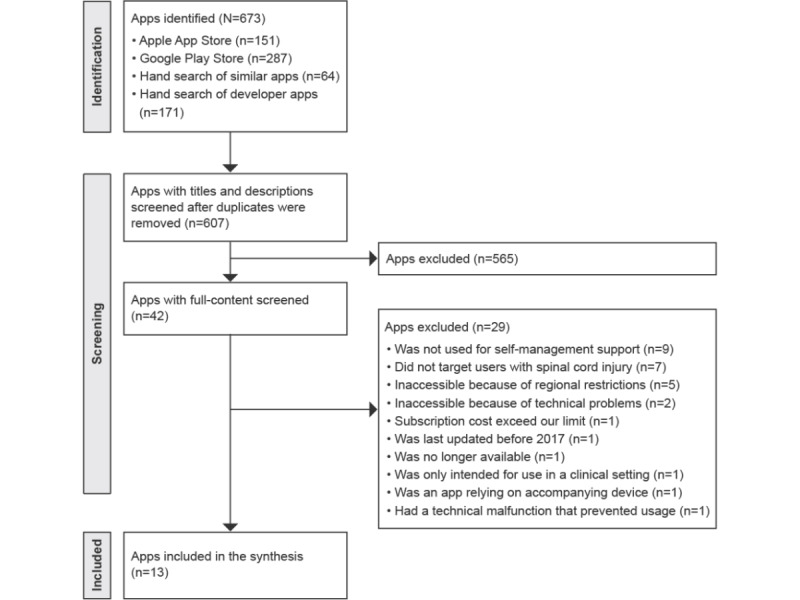
PRISMA (Preferred Reporting Items for Systematic Reviews and Meta-Analyses) flowchart of the app store search, selection, and inclusion process.

**Figure 2:**
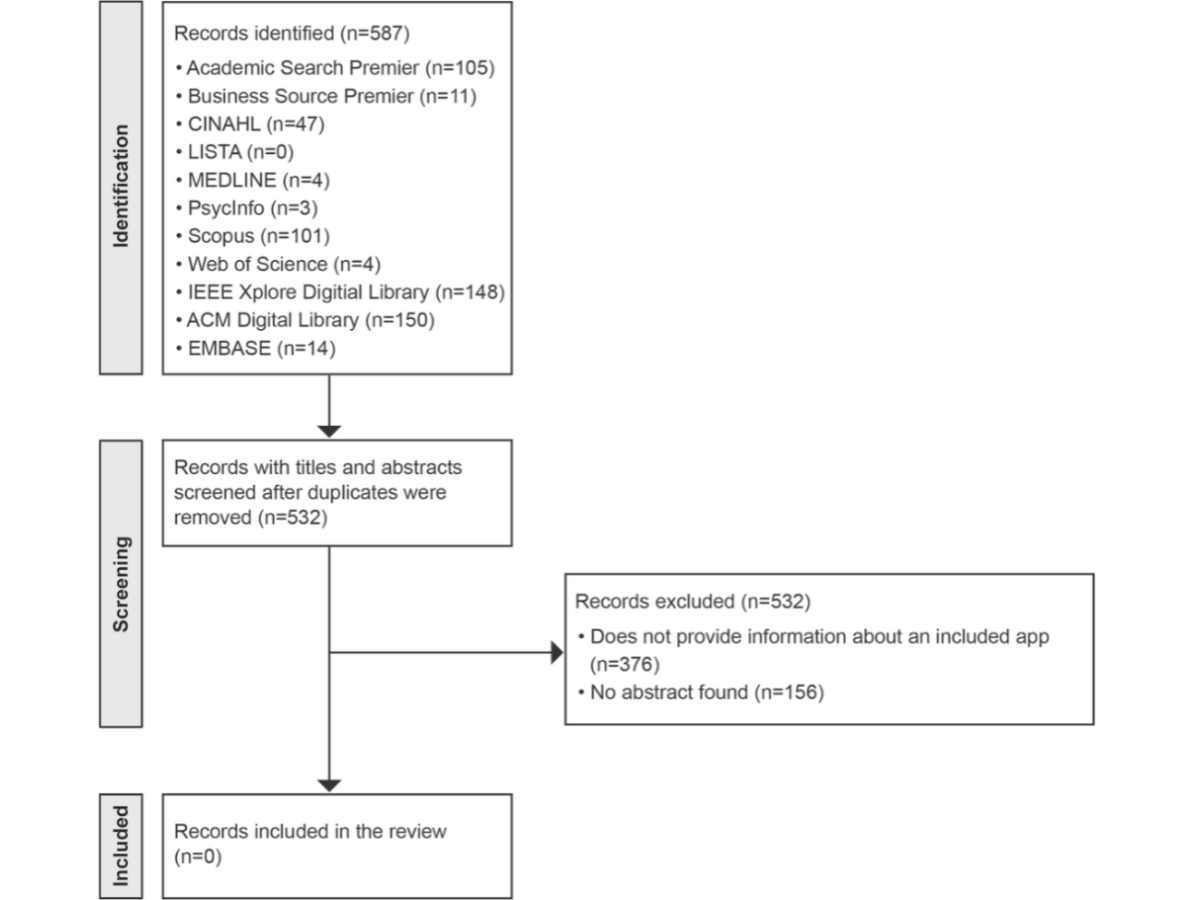
PRISMA (Preferred Reporting Items for Systematic Reviews and Meta-Analyses) flowchart of the literature search, selection, and inclusion process.

A total of 13 apps were included in the final synthesis, and no publications describing them were identified. Figures 1 and 2 detail the corresponding methodological processes (see Multimedia Appendix 2 for the PRISMA checklist).

### App Characteristics

#### Overview

The 13 identified apps are summarized in Multimedia Appendix 3. They were launched between 2013 and 2021, with the majority (9/13, 69%) released from 2019 onward [35-43] (Table 1). These apps mainly originated from the United States (8/13, 62%) [35,38-41,44-46], followed by Australia (2/13, 15%) [37,42] and 1 (1/13, 8%) each from Belarus [47], France [36], and Italy [43]. They were mainly published under the Health category (8/13, 62%) [37-40,42,43,46,47], followed by 1 (1/13, 8%) app each under the Tools [35], News and magazines [44], Sports [45], Communication [36], and Lifestyle [41] categories across both app stores. The identified apps supported 14 languages, with English having full support due to the language eligibility requirement, followed by French (4/13, 31%); German and Italian (3/13, 23%); Spanish and Portuguese (2/13, 15%); and several other European languages, Turkish, and Japanese (1/13, 8%). The identified apps were available in 72 countries, covering all 6 major world regions and 16 (73%) of the 22 subregions globally [48]. They were unavailable in countries in central Asia, middle Africa, eastern Africa, Melanesia, Polynesia, and Micronesia. Where reported (8/13, 62%) [35-39,44,45,47], 15% (2/13) of the identified apps had between 500,001 and 1,000,000 [35,47], 1001 and 5000 [36,44], 101 and 1000 [37,45], and 6 and 50 [38,39] downloads each. The number of downloads for the 5 Apple App Store apps was not reported. Target user ages were not reported, but most apps had content deemed appropriate for all ages (9/13, 69%) [35-40,44,45,47] and a few for adults only (4/13, 31%) [41-43,46].

**Table 1:** Characteristics of the 13 identified self-management apps for spinal cord injury by frequency.

Characteristic			Apps, n (%)
**Year of launch**			
	2020 [35-37,39]	4 (31)	
	2019 [38,40-42]	4 (31)	
	2013 [46,47]	2 (15)	
	2017 [44,45]	2 (15)	
	2021 [43]	1 (8)	
**Country of origin**			
	United States [35,38-41,44-46]	8 (62)	
	Australia [37,42]	2 (15)	
	Belarus [47]	1 (8)	
	France [36]	1 (8)	
	Italy [43]	1 (8)	
**Regional availability**			
	Australia and New Zealand [35-47]	13 (100)	
	Northern America [35-47]	13 (100)	
	Western Europe [35-47]	13 (100)	
	Northern Africa [35,36,38-47]	12 (92)	
	Northern Europe [35,36,38-47]	12 (92)	
	South America [35,36,38-47]	12 (92)	
	Central America [35,38-47]	11 (85)	
	Southern Africa [35,38-47]	11 (85)	
	Western Africa [35,38-47]	11 (85)	
	Eastern Asia [35,38-47]	11 (85)	
	Southeastern Asia [35,38-47]	11 (85)	
	Southern Asia [35,38-47]	11 (85)	
	Western Asia [35,38-47]	11 (85)	
	Eastern Europe [35,38-47]	11 (85)	
	Southern Europe [35,38-47]	11 (85)	
	Caribbean [40-42,46]	4 (31)	
**Supported languages**			
	English [35-47]	13 (100)	
	French [35,36,38,47]	4 (31)	
	Italian [35,43,47]	3 (23)	
	German [35,36,47]	3 (23)	
	Spanish [35,47]	2 (15)	
	Portuguese [35,47]	2 (15)	
	Czech [47]	1 (8)	
	Danish [47]	1 (8)	
	Greek [47]	1 (8)	
	Japanese [35]	1 (8)	
	Russian [47]	1 (8)	
	Slovak [47]	1 (8)	
	Swedish [47]	1 (8)	
	Turkish [47]	1 (8)	
**Operating system**			
	Android [35-39,44,45,47]	8 (62)	
	iOS [40-43,46]	5 (38)	
**App store category**			
	Health and fitness [37-40,42,47]	6 (46)	
	Medical [43,46]	2 (15)	
	Tools [35]	1 (8)	
	News and magazines [44]	1 (8)	
	Sports [45]	1 (8)	
	Communication [36]	1 (8)	
	Lifestyle [41]	1 (8)	
**Year of last update**			
	2020 [39-41,44,45]	5 (38)	
	2022 [35-37,43,46]	5 (38)	
	2019 [38,42,47]	3 (23)	
**Cost to download (US $)**			
	0.00 [35,37,39,41-44,46,47]	6 (46)	
	≤5.00 [36,38,40]	3 (23)	
	≤10.00 [39,47]	2 (15)	
	≤15.00 [45]	1 (8)	
	≤20.00 [44]	1 (8)	
**In-app purchases**			
	No [35,37-43,47]	9 (69)	
	Yes [36,44-46]	4 (31)	
**Downloads**			
	NR^a^ [40-43,46]	5 (38)	
	≥500,000 [35,47]	2 (15)	
	≥1000 [36,44]	2 (15)	
	≥100 [45]	1 (8)	
	≥500 [37]	1 (8)	
	≥10 [38]	1 (8)	
	≥5 [39]	1 (8)	

^a^NR: not reported.

#### Platforms and Functionality

Of the 13 apps included in this study, 10 (77%) used the Android operating system [49-56] and 4 (31%) used iOS [57-59]. Of the 13 apps, 2 (15%) used the Android operating system only [49,54], 3 (23%) used iOS only [57-59], and 6 (46%) used both Android and iOS [50-53,55,56,60-65]. The earliest operating system versions supported by the identified apps were Android version 4 (5/13, 38%) [38,39,44,45,47] and iOS version 9 (1/13, 8%) [40]. The identified apps had a file size ranging from 3 MB to 117 MB, with the average size being 31 (SD 35.18) MB for Android and 47 (SD 28.15) MB for iOS. Of the identified apps, half (7/13, 54%) had a tablet version [35,37,39,40,44-46]. Most of the identified apps (9/13, 69%) were functional without an internet connection [35-40,42,43,47]. The identified apps had no discernible way for users to access their data and freely export their data. Of the 13 apps, 1 (8%) incorporated gamification [42], 2 (15%) included assistive videos [42,47], and 3 (23%) provided music or audio [39,42,47].

Very few of the included apps (3/13, 23%) had a wide range of features. Of the 13 apps, only 2 (15%) offered tracking functionality for physical activity [37,42], and 1 (8%) app had features to support mindfulness and deep breathing exercises as a form of sleep therapy [39]. None of the apps provided a platform for peer support, though 1 (8%) of the 13 apps did provide a connection to a support professional [37]. No apps provided features for psychoeducation, journaling, goal setting, habits, or chatbot interactions.

#### Privacy and Support

Most of the identified apps (8/13, 62%) provided accessible privacy policies [35-41,43]. The remaining apps either had broken web page links to the privacy policies [42,44-46] or provided none [47]. When provided, privacy policies were accessible from the app store and app (4/13, 31%) [37,39-41] and app store alone (8/13, 62%) [35-41,43]. No clarification on personal data processing and data security measures was provided. Of the 13 apps, 1 (8%) [37] was identified as sharing personal health information as it connected users to health care providers. No deidentified, anonymized, or aggregated data were seemingly shared by the identified apps, and they did not claim to meet any regulation targeting data privacy or security such as the Health Insurance Portability and Accountability Act of 1996 in the United States. Data collection was mandatory for the identified apps. However, it was difficult to distinguish between optional and mandatory data collection. As a result, this distinction was not included in the synthesis. See Table 2 for more information.

**Table 2:** User data collected by the 13 identified self-management apps for spinal cord injury by purpose.

Data collected	Purpose	Apps, n (%)
Network connections	Analytics [35] and unknown [36-39,44,45,47]	8 (62)
Email address	Analytics [35-37,39], app functionality [36-39,41], developer communications [36,37,39], security and compliance [36,37,39], personalization [36,39], and account management [36,39]	6 (46)
App interactions	Analytics [35,36], app functionality [36], developer communications [36], and unknown [38,41]	4 (31)
Contact list	App functionality [37,41] and personalization [37]	4 (31)
Device ID	Analytics [35,37,38] and security and compliance [36-38]	4 (31)
IP address	Analytics [37-40] and security and compliance [38]	4 (31)
Credit card information	App functionality [37] and unknown [38,41]	3 (23)
Location	App functionality [37], personalization [37], and unknown [39]	2 (15)
Log data	Analytics [36,37,40]	3 (23)
Name	App functionality [37,38,41] and personalization [37,38]	3 (23)
Phone number	Analytics [36], app functionality [36], developer communications [36,37], security and compliance [36,37,41], personalization [36,41], account management [36], and unknown [36]	3 (23)
Age	Analytics [37], app functionality [37,38], and personalization [37,38]	2 (15)
Crash logs	Analytics [35,36], app functionality [36], and developer communications [36]	2 (15)
Date of birth	Analytics [37,41], app functionality [37], and personalization [37,41]	2 (15)
Device type	Analytics [37,41] and security and compliance [37,41]	2 (15)
Diagnostics	Analytics [35,36], app functionality [36], and developer communications [36]	2 (15)
Postal address	Analytics [37], app functionality [37], developer communications [37], and personalization [41]	2 (15)
User ID	Analytics [35,36], app functionality [36], developer communications [36], security and compliance [36], personalization [36], and account management [36]	2 (15)
Gender	Analytics [41] and personalization [41]	1 (8)
In-app search history	Analytics [35]	1 (8)
Other user-generated content	Analytics [35] and app functionality [35]	1 (8)
Statistics on page views	Analytics [37]	1 (8)

Most apps provided support options (12/13, 92%) and used email (6/13, 46%) [35,36,39,41,42,47]; website-based information (6/13, 46%) [36,37,40,42,44,45]; tutorials, guides, or manuals (6/13, 46%) [36,37,40,44-46]; frequently asked questions (3/13, 23%) [36,37,41]; phone (2/13, 15%) [35,42]; web chat (1/13, 8%) [35]; contact forms (1/13, 8%) [42]; and social media platforms (1/13, 8%) [37] for this purpose. Of the 13 apps, 4 (31%) provided 2 options [40,41,44,45], 3 (23%) provided 4 options [36,37,42] and 1 option [39,46,47], and 1 (8%) provided 3 options [35].

#### Cost

Of the 13 apps, 5 (38%) did not request payment [35,37,41-43] and the remaining 8 (62%) accepted payment [36,38-40,44-47] to download [36,38-40,44,45,47], while 4 (31%) accepted payment for one-time or recurring in-app purchases to access full features [36,44-46]. The cost to download ranged from US $1.99 to US $19.99.

#### Developers

Developers were mainly for-profit companies (10/11, 91%) [49,50,52-60,62-65] except for 1 (9%) nonprofit company [51,61]. Developers published 246 apps across both app stores [49-65], with most of them being published in Google Play Store (137/246, 55.7%) [49-56] and the remainder in the Apple App Store (109/246, 44.3%) [57-65] (Table 3). Half of the developers focused on publishing health-related apps (6/11, 55%) [49,53-55,57,59,63,64], and the others published apps across a wide range of categories in the app store [50-52,56,58,60-62,65]. The Paralyzed Veterans of America [51,61] published 3 (23%) of the 13 identified apps [44-46]; other developers (10/11, 91%) published 1 each [35-43,47].

**Table 3:** Characteristics of the developers of the identified self-management apps for spinal cord injury (N=11).

Developer name and citation	Country	Company type	App store categories with published apps
DSN Inc [49]	Belarus	For-profit company	Health and fitness and puzzle
Google LLC [50,60]	United States	For-profit company	Auto and vehicles, books and reference, business, communication, education, entertainment, finance, health and fitness, libraries and demo, lifestyle, medical, music and audio, news and magazines, personalization, photo and video, photography, productivity, simulation, social, tools, travel and local, utilities, utilities, video players and editors, and weather
Paralyzed Veterans of America [51,61]	United States	Nonprofit company	Business, medical, news and magazines, and sports
JIB Smart Home [52,62]	France	For-profit company	Communication, house and home, and style
Maslow For People and Ilya Thai [53,63]	Australia	For-profit company	Health and fitness
Kinnereth LLC App Dev [54]	United States	For-profit company	Health and fitness
Injectful LLC [55,64]	United States	For-profit company	Health and fitness
Cordilac LC [57]	United States	For-profit company	Health and fitness
iAccess Innovations [56,65]	United States	For-profit company	Lifestyle
Monster Hub [58]	Australia	For-profit company	Business, education, entertainment, games, health and fitness, lifestyle, productivity, shopping, social networking, sports, and travel
Giorgio Lofrese [59]	Italy	For-profit company	Medical

### Characteristics of Approaches Providing mHealth Self-Management Support for SCI

All self-management tasks were supported by the identified apps (Table 4). Role management received the most support (11/13, 85%) [35-38,40-42,44-47], followed by medical (7/13, 54%) [37,38,40,42,43,45,47] and emotional (4/13, 31%) [39,42,44,45] management (Table 5). Support for a single task was most common (6/13, 46%) [35,36,39,41,43,46], followed by 2 (5/13, 38%) [37,38,40,44,47] and 3 (2/13, 15%) [42,45] tasks. When combined, role and medical management were most often supported together (4/13, 31%) [37,38,40,47], followed by role and emotional management (1/13, 8%) [44].

**Table 4:** Identified self-management apps for spinal cord injury by self-management approach (N=13).

App name and citation	Self-management focus area	Relevant self-management tasks	Relevant self-management skills	Relevant self-management support components
Pilates [47]	Physical activity promotion	Role management and medical management	Action planning	Training and rehearsal for practical self-management activities
Action Blocks [35]	Activities of daily living	Role management	Resource use	Training and rehearsal for practical self-management activities, training and rehearsal for everyday activities, and lifestyle advice and support
PN–Paraplegia News [44]	Health literacy and activities of daily living	Role management and emotional management	Problem-solving, action planning, and resource use	Information about the condition and or its management, information about available resources, and lifestyle advice and support
SNS Digital [45]	Physical activity promotion, health literacy, and activities of daily living	Role management, emotional management, and medical management	Problem-solving, action planning, and resource use	Information about available resources and lifestyle advice and support
JIB CALLS [36]	Activities of daily living	Role management	Resource use	Training and rehearsal for everyday activities
Disability Care App [37]	Therapeutic exercise and activities of daily living	Role management and medical management	Action planning, maintaining patient-provider partnership, and problem-solving	Practical support with adherence (medication or behavioral), training and rehearsal for practical self-management activities, and training and rehearsal to communicate with health care professionals
Dietitian’s Tools [38]	Medicating and dieting	Role management and medical management	Action planning	Training and rehearsal for practical self-management activities and training and rehearsal to communicate with health care professionals
Injectful [39]	Pain management	Emotional management	Problem-solving	Training and rehearsal for psychological strategies
PVA ePubs [46]	Health literacy and activities of daily living	Role management	Resource use and action planning	Information about available resources and lifestyle advice and support
AccessiRep [40]	Physical activity promotion	Role management and medical management	Problem-solving and action planning	Training and rehearsal for practical self-management activities
iAccess Life–Accessibility [41]	Mobility	Role management	Resource use and action planning	Lifestyle advice and support, social support, and information about available resources
Neuro Therapy [42]	Therapeutic exercise and physical activity promotion	Role management, emotional management, and medical management	Action planning and maintaining patient-provider partnership	Training and rehearsal for practical self-management activities, practical support with adherence (medication or behavioral), provision of easy access to advice or support when needed, and social support
Spine Fine [43]	Therapeutic exercise and physical activity promotion	Medical management	Action planning	Training and rehearsal for practical self-management activities, monitoring of the condition with feedback, and provision of easy access to advice or support when needed

**Table 5:** Characteristics of approaches to self-management support for spinal cord injury by frequency (N=13).

Characteristic		Apps, n (%)
**Supported self-management tasks**		
	Role management [35-38,40-42,44-47]	11 (85)
	Emotional management [39,42,44,45]	4 (31)
	Medical management [37,38,40,42,43,45,47]	7 (54)
**Supported self-management skills**		
	Action planning [37,38,40-47]	10 (77)
	Resource use [35,36,41,44-46]	6 (46)
	Problem-solving [37,39,40,44,45]	5 (38)
	Maintaining patient-provider partnership [37,42]	2 (15)
**Incorporated self-management support components**		
	Training and rehearsal for practical self-management activities [35,37,38,40,42,43,47]	7 (54)
	Lifestyle advice and support [35,41,44-46]	5 (38)
	Information about available resources [41,44-46]	4 (31)
	Social support [41,42]	2 (15)
	Practical support with adherence (medication or behavioral) [37,42]	2 (15)
	Provision of easy access to advice or support when needed [42,43]	2 (15)
	Training and rehearsal for everyday activities [35,36]	2 (15)
	Training and rehearsal to communicate with health care professionals [37,38]	2 (15)
	Monitoring of the condition with feedback [43]	1 (8)
	Training and rehearsal for psychological strategies [39]	1 (8)
**Targeted self-management focus areas**		
	Activities of daily living [35-37,44-46]	6 (46)
	Physical activity promotion [40,42,43,45,47]	5 (38)
	Health literacy [44-46]	3 (23)
	Therapeutic exercise [37,42,43]	3 (23)
	Mobility [41]	1 (8)
	Medicating and dieting [38]	1 (8)
	Pain management [39]	1 (8)

Most self-management skills were supported by the identified apps (4/6, 67%; Table 5). *Action planning* received support from most of the identified apps (10/13, 77%) [37,38,40-47], whereas *maintaining patient-provider partnership* received the least support (2/13, 15%) [37,42]. Support for a single skill was most common (6/13, 46%) [35,36,38,39,43,47], followed by 2 (4/13, 31%) [40-42,46] and 3 (3/13, 23%) [37,44,45] skills.

Incorporated self-management support components included 71% (10/14) of the Practical Reviews in Self-Management Support components (Table 5). The top 3 components were incorporated more than the average number of times and accounted for 85% (11/13) of the identified apps. Information regarding the condition and its management, including equipment details, specific clinical action plans, rescue medications, and regular clinical reviews, was not included. Of the 13 apps, 6 (46%) incorporated 1 component [35,36,38,39,43,47], 4 (31%) incorporated 2 components [40-42,46], and 3 (23%) incorporated 3 components [37,44,45].

The adopted approaches to providing self-management support mainly focused on activities of daily living, physical activity promotion, health literacy, and therapeutic exercise (Table 5). Mobility, medicating and dieting, and pain management were targeted to a lesser extent.

### MARS Evaluation

The mean MARS score for the 13 identified apps was 3.86 (SD 0.54), with a maximum of 4.52 for Action Blocks [35] and a minimum of 2.72 for AccessiRep [40], indicating good overall quality (Table 6). On average, the best-rated section was functionality (mean 4.56, SD 0.33), followed by information quality (mean 3.71, SD 0.78), aesthetics (mean 3.62, SD 0.91), engagement (mean 3.54, SD 0.61), app subjective quality (mean 3.39, SD 0.98), and app specific quality (mean 2.73, SD 0.97). The mean MARS score for the apps from Google Play and Apple App Store were 3.84 (SD 0.47; 8/13, 61%) and 3.88 (SD 0.71; 5/13, 39%), respectively. The interrater reliability as assessed by the intraclass correlation coefficient was 0.80 (95% CI 0.49-0.94), indicating a good level of agreement in the scoring between the 2 raters (AV and MD) [66].

**Table 6:** Mobile App Rating Scale scores (overall score and 4 subscales) of the 13 identified self-management apps for spinal cord injury.

App name and citation; developer and citation	Overall score	Engagement score	Functionality score	Aesthetics score	Information quality score
Action Blocks [35]; Google LLC [50,60]	4.52	4.40	4.75	4.50	4.42
PVA ePubs [46]; Paralyzed Veterans of America [51,61]	4.48	3.50	4.88	4.83	4.70
Neuro Therapy [42]; Monster Hub [58]	4.44	4.30	4.38	4.50	4.60
Disability Care App [37]; Maslow For People [53,63]	4.19	4.20	4.63	4.67	3.25
JIB CALLS [36]; JIB Smart Home [52,62]	3.98	3.80	4.88	4.00	3.25
Injectful [39]; Injectful LLC [55,64]	3.93	3.50	4.63	4.00	3.60
iAccess Life–Accessibility [41]; iAccess Innovations [56,65]	3.92	3.80	4.88	3.50	3.50
PN–Paraplegia News [44]; Paralyzed Veterans of America [51,61]	3.85	3.40	4.50	3.00	4.50
SNS Digital [45]; Paralyzed Veterans of America [51,61]	3.85	3.40	4.50	3.00	4.50
Spine Fine [43]; Giorgio Lofrese [59]	3.83	3.60	4.38	3.83	3.50
Dietitian’s Tools [38]; Kinnereth LLC App Dev [54]	3.46	2.80	4.88	2.67	3.50
Pilates [47]; DSN Inc [49]	2.96	3.10	4.25	2.17	2.33
AccessiRep [40]; Cordilac LC [57]	2.72	2.20	3.75	2.33	2.58

Regarding *engagement*, most of the identified apps were deemed sufficiently customizable, interactive, and focused on the targeted audience [35-38,41,42,47], as indicated by the minimum acceptability score of 3.0 established by the MARS. Nonetheless, several apps were rated lower due to lacking sufficient settings for customization [39,40,43-47]. In terms of *functionality*, the identified apps were generally easy to use and had good navigation and gestural design. Similarly, the identified apps received high scores for aesthetics, as they featured appealing graphic designs, color schemes, and layouts that were consistent throughout. Concerning *information quality*, most apps had accurate descriptions and presented enough information of good quality. However, low or no scores were noted regarding the presence of specific, measurable, and achievable goals as well as an evidence base supported by scientific research or expert-reviewed guidelines for the app’s content and functionality.

## Discussion

### Principal Findings and Comparison With Prior Work

This study identified 13 self-management support apps for SCI available on the Google Play Store and Apple App Store that meet this study’s inclusion criteria. No scientific literature was found that focused on designing, describing, evaluating, piloting, implementing, or improving them. This emphasizes the critical shortage of evidential support in literature for these apps and underscores the uncertainty around their quality and effectiveness. Consequently, it becomes challenging to confidently recommend any of them or identify which aspects require improvement. Unsurprisingly, none of the apps were identified by an earlier systematic literature review with similar inclusion criteria [67]. This highlights the need for greater collaboration between researchers and developers to improve the dissemination and availability of theory- and evidence-based self-management support apps for SCI.

### Self-Management Support Apps for SCI

This study identified 11 self-management support apps launched on the Google Play Store and Apple App Store between 2017 and 2021, similar in number to the 11 apps identified by the earlier systematic literature review [67]. As all apps were unique, a total of 22 self-management support apps for SCI were available during this period. The United States continues to contribute most of the apps identified, which may result in a potential bias toward the cultural, social, and demographic context of the country and may not fully capture the diverse needs and preferences of users from other regions. Although apps from Canada, the Netherlands, Switzerland, and Thailand were discussed in the literature, no similar apps from these countries were found on app distribution platforms. According to this review, Australia, Belarus, and France were also represented for the first time. The number of downloads for Apple App Store apps was not reported, limiting our understanding of the popularity and reach of these apps. It was noted that most apps did not require an internet connection, which is beneficial for areas with poor or no internet connectivity.

### mHealth Self-Management Support Approaches in SCI

This study identified self-management focus areas that align with those found in the earlier systematic literature review [67]. These included physical activity promotion, pain management, therapeutic exercise, medication, and dieting. The identified apps in this review also supported all self-management tasks; emotional management received the least amount of support, albeit more than in the earlier review. While the support for role management was slightly less but almost equal to medical management in the earlier review, the identified apps in this review provided more resources in support of role management. The general trend in the coverage of support for self-management tasks remains consistent with the earlier review. However, the apps identified in this study provide more comprehensive support for these tasks compared to the previous review. Unlike in the earlier review, *decision-making* and *self-tailoring* self-management skills were unsupported by apps identified in this study. They were also 2 (50%) of the top 4 skills supported by mHealth covered in literature. The trend in the coverage of support for self-management skills and support activities remains consistent with the earlier review, but the identified apps provide less coverage for these skills and activities.

### Quality of Self-Management Support Apps for SCI

Similar to findings from this study, recent reviews on self-management support apps for food allergies or intolerances [68], depression [69], rare diseases [70], and diabetes (3.42) [71] also report that the overall quality of self-management support apps assessed are generally acceptable. App *functionality* also received the highest ratings across these studies, which suggests that self-management support apps tend to be user-friendly. A higher *engagement* score was reported by this study compared to the other aforementioned studies, suggesting that self-management support apps for SCI are more engaging on average. *Aesthetics* received high mean scores across the studies as well, suggesting that self-management support apps across varying health conditions had appealing graphic designs, color schemes, and layouts. *Information quality* scores were also similar among these studies.

### Implications for Future Practice and Research

More apps that provide comprehensive self-management support for SCI are needed. Adopting new development methods could help reduce barriers to development, such as time, cost, and securing scarce expertise. For example, low-code development platforms provide prebuilt templates and drag-and-drop interfaces, allowing apps to be created with minimal hand-coding. In addition, customizing white-label solutions can further streamline the development process. Adopting an iterative co-design approach involving key stakeholders, such as persons with SCI, health professionals, app developers, carers, and researchers, would also be key for app success. This approach could also incorporate validation studies with health professionals, efficacy and effectiveness studies through collaborations with researchers, and quality evaluations with a wide range of key stakeholders using tools such as MARS [72].

Complying with best practices should also be considered. App store categorization should be more consistent to improve the discoverability and accessibility for potential users. Privacy notices must be accessible via app stores so users can make an informed decision at the point of download and in the app. Providing information about the required device permissions is also useful.

It is critical for future research to provide a supportive evidence base for the development of high-quality self-management support apps for SCI, as none appears to exist. Investigations using app use data are needed to adequately determine their feasibility. App download counts are insufficient as they only indicate awareness of the app and potentially the initial willingness to use it. Further investigation into the data collection practices of these apps is warranted due to the challenges in distinguishing between optional and mandatory data collection and the associated concerns about the potential data privacy risks and implications. Given the high turnover of available apps on these platforms, future systematic searches in app stores need to continuously monitor and capture this information to increase the awareness of available high-quality self-management support apps that could benefit people with SCI. Persons with SCI, their carers, and related health professionals should be asked to recommend self-management support apps for consideration. Searching a wider range of mobile app digital distribution platforms could also be beneficial for identifying more self-management support apps. Furthermore, it could be beneficial to validate the reported findings with datasets from these other platforms. Research should also consider supplementing the MARS with other instruments for a more comprehensive assessment and description of apps, such as the app evaluation model by the American Psychiatric Association [73], behavior change technique taxonomy [23], and IMS Institute for Healthcare Informatics app functionality scale [22].

### Limitations

Although the most popular by a wide margin, only 2 of the many mobile app digital distribution platforms (eg, Microsoft Store, BlackBerry World, Huawei, and AppGallery) were searched, and some eligible apps could have been overlooked. The app store search keywords were broad but also limited to ensure that screening was feasible for the small team, and it cannot be guaranteed that all eligible apps were retrieved. Using a proprietary application programming interface potentially imposes limitations in terms of transparency relating to search accuracy, comprehensiveness, and documentation, which could affect the reproducibility of this study. Although unlikely, it is acknowledged that some eligible apps might have been missed due to the English language restriction. Not considering all app versions across both platforms could have biased the results, but this was unlikely in this synthesis as the identified apps were identical versions and were almost indistinguishable beyond differences imposed by the platform. Assessing identified apps in the English language could have also biased the results as other translations might not provide a similar user experience. While the inclusion of privacy policies was noted, analyzing their content and drawing conclusions about their conciseness, transparency, intelligibility, and accessibility was beyond the scope of this synthesis. The MARS neglects important aspects of app quality, such as data privacy, security, and accessibility, but provides sufficient scope with its 5 dimensions for the purposes of this initial overview of self-management support apps for SCI. Due to the exclusion of several inapplicable items from the mean score of the *information* dimension, comparisons between apps with and without complete data, especially for this dimension, should be cautiously interpreted. As mobile app digital distribution platforms constantly undergo swift changes, it is acknowledged that these findings provide a snapshot. The identified apps may have been updated or no longer available, their content may have been modified, or new mobile apps may have been developed since this systematic search was conducted, and this can limit the generalizability of this study’s findings. Furthermore, these findings may not be generalizable to all self-management support apps for SCI, as the inclusion criteria were specific to certain types of apps, including those available in English and in certain app stores, such as Google Play Store and Apple App Store.

### Conclusions

This study contributes to a broader understanding of mHealth self-management support tools for SCI by identifying and evaluating 13 self-management support apps for SCI. While these apps had good overall quality as measured by the MARS assessment, greater collaboration between developers and researchers is needed to improve the dissemination and availability of theory- and evidence-based self-management support apps for SCI. Failure to adopt this approach could limit the potential impact of these apps and result in missed opportunities to improve self-management support for people living with SCI. Furthermore, exploring new app development methods, such as using low-code platforms, could help promote stakeholder inclusivity in the development process and reduce barriers to app development, including time, cost, and expertise.

## Appendix

Multimedia Appendix 1 Search concepts and terms and search strategies for queried databases.

Multimedia Appendix 2 PRISMA (Preferred Reporting Items for Systematic Reviews and Meta-Analyses) checklist.

Multimedia Appendix 3 Characteristics of the 13 identified self-management apps for spinal cord injury.

## Abbreviations

MARS: Mobile App Rating Scale
mHealth: mobile health
PRISMA: Preferred Reporting Items for Systematic Reviews and Meta-Analyses
SCI: spinal cord injury
