# Supplementary material for: Self-Management Support Apps for Spinal Cord Injury: Results of a Systematic Search in App Stores and Mobile App Rating Scale Evaluation
Source: JMIR Mhealth Uhealth. 2024 Dec 19;12:e53677. doi: 10.2196/53677 (PMC11695972; doi:10.2196/53677)
Supplement: Multimedia Appendix 1 [file mhealth_v12i1e53677_app1.docx]

## Multimedia Appendix 1

**Search concepts and terms, and search strategies for queried databases**

Table 1. Search concepts and terms, and search strategies for queried digital distribution platforms

| **Concepts** | **Terms** | **Example JSON (JavaScript Object Notation) syntax** |
| --- | --- | --- |
| spinal cord injury | spinal cord, paraplegia, paraplegias, paraplegic, paraplegics, paraparesis, quadriplegia, quadriplegias, quadriplegic, quadriplegics, quadraparesis, tetraplegia, tetraplegias, tetraplegic, tetraplegics, tetraparesis, myelopathy, paralysis | {  "query":  {  "query_params":  {  "num":50,   "full_text_term": " paraplegia OR paraplegias OR paraplegic OR paraplegics OR paraparesis OR quadriplegia OR quadriplegias ",  "include_full_text_desc":true,  "include_developer":true  }  } } |

Table 2. Search concepts and terms, and search strategies for queried bibliographic databases

| **Academic Search Premier** | | |
| --- | --- | --- |
| **#** | **Query** | **Results** |
| S34 | S3 AND S33 (Document Type: Article, Book Chapter, Case Study, Proceeding, Report; Language: English) | 105 |
| S33 | S3 AND S44 | 141 |
| S32 | S4 OR S5 OR S6 OR S7 OR S8 OR S9 OR S10 OR S11 OR S12 OR S13 OR S14 OR S15 OR S16 OR S17 | 157 |
| S31 | S3 AND S17 | 5 |
| S30 | S3 AND S16 | 21 |
| S29 | S3 AND S15 | 2 |
| S28 | S3 AND S14 | 0 |
| S27 | S3 AND S13 | 0 |
| S26 | S3 AND S12 | 12 |
| S25 | S3 AND S11 | 0 |
| S24 | S3 AND S10 | 17 |
| S23 | S3 AND S9 | 0 |
| S22 | S3 AND S8 | 0 |
| S21 | S3 AND S7 | 6 |
| S20 | S3 AND S6 | 6 |
| S19 | S3 AND S5 | 0 |
| S18 | S3 AND S4 | 4 |
| S17 | TX ("Spine Fine" OR "Giorgio Lofrese") | 6 |
| S16 | TX ("Neuro Therapy" OR "Monster Hub" OR "combined wellness solutions") | 27 |
| S15 | TX ("iAccess Life" OR "Rate & Review Accessibility" OR "iAccess Innovations") | 2 |
| S14 | TX (AccessiRep OR "Automatic Repetition Counter" OR "Cordical LC") | 0 |
| S13 | TX ("PVA ePubs" AND "Paralyzed Veterans of America") | 3 |
| S12 | TX (Injectful OR "Ehren Nelson" OR "Nicholas Barnes") | 15 |
| S11 | TX ("Dietitian's Tools" OR "Kinnereth LLC App Dev" OR "Grace Nutrition Consulting") | 0 |
| S10 | TX ("Maslow For People" OR "Maslow Pty") | 17 |
| S9 | TX ("JIB CALLS" OR "JIB Smart Home") | 8 |
| S8 | TX ("SNS Digital" AND "Paralyzed Veterans of America") | 391 |
| S7 | TX ("Paraplegia News" AND "Paralyzed Veterans of America") | 9 |
| S6 | TX ("Action Blocks" AND google) | 7 |
| S5 | TX ("Accessible Pakistan" OR SoftMining) | 0 |
| S4 | TX (Pilates AND DSN) | 4 |
| S3 | S1 OR S2 | 15,646,865 |
| S2 | TX (self-management OR self-care OR self-regulation OR self-monitoring) | 177,709 |
| S1 | TX (app* OR "mobile device" OR application* OR smartphone* OR application* OR iOS OR android OR tablet* OR "wireless technology" OR "wireless technologies" OR mhealth) | 15,613,611 |
|  |  |  |
| **Business Source Premier** | | |
| **#** | **Query** | **Results** |
| S34 | S3 AND S33 (Publication Type: Academic Journal, Book; Document Type: Article, Case Study, Company Report, Proceeding, Report, Working Paper; Language: English) | 11 |
| S33 | S3 AND S44 | 95 |
| S32 | S4 OR S5 OR S6 OR S7 OR S8 OR S9 OR S10 OR S11 OR S12 OR S13 OR S14 OR S15 OR S16 OR S17 | 103 |
| S31 | S3 AND S17 | 0 |
| S30 | S3 AND S16 | 10 |
| S29 | S3 AND S15 | 2 |
| S28 | S3 AND S14 | 0 |
| S27 | S3 AND S13 | 0 |
| S26 | S3 AND S12 | 7 |
| S25 | S3 AND S11 | 0 |
| S24 | S3 AND S10 | 14 |
| S23 | S3 AND S9 | 0 |
| S22 | S3 AND S8 | 0 |
| S21 | S3 AND S7 | 1 |
| S20 | S3 AND S6 | 1 |
| S19 | S3 AND S5 | 1 |
| S18 | S3 AND S4 | 2 |
| S17 | TX ("Spine Fine" OR "Giorgio Lofrese") | 1 |
| S16 | TX ("Neuro Therapy" OR "Monster Hub" OR "combined wellness solutions") | 11 |
| S15 | TX ("iAccess Life" OR "Rate & Review Accessibility" OR "iAccess Innovations") | 2 |
| S14 | TX (AccessiRep OR "Automatic Repetition Counter" OR "Cordical LC") | 190 |
| S13 | TX ("PVA ePubs" AND "Paralyzed Veterans of America") | 0 |
| S12 | TX (Injectful OR "Ehren Nelson" OR "Nicholas Barnes") | 9 |
| S11 | TX ("Dietitian's Tools" OR "Kinnereth LLC App Dev" OR "Grace Nutrition Consulting") | 5 |
| S10 | TX ("Maslow For People" OR "Maslow Pty") | 15 |
| S9 | TX ("JIB CALLS" OR "JIB Smart Home") | 6 |
| S8 | TX ("SNS Digital" AND "Paralyzed Veterans of America") | 213 |
| S7 | TX ("Paraplegia News" AND "Paralyzed Veterans of America") | 1 |
| S6 | TX ("Action Blocks" AND google) | 2 |
| S5 | TX ("Accessible Pakistan" OR SoftMining) | 1 |
| S4 | TX (Pilates AND DSN) | 3 |
| S3 | S1 OR S2 | 8,166,002 |
| S2 | TX (self-management OR self-care OR self-regulation OR self-monitoring) | 50,672 |
| S1 | TX (app* OR "mobile device" OR application* OR smartphone* OR application* OR iOS OR android OR tablet* OR "wireless technology" OR "wireless technologies" OR mhealth) | 8,157,324 |
|  |  |  |
| **CINAHL** | | |
| **#** | **Query** | **Results** |
| S34 | S3 AND S33 (Publication Type: Book, Book Chapter, Brief Item, Case Study, Clinical Trial, Doctoral Dissertation, Journal Article, Masters Thesis, Meta Analysis, Meta Synthesis, Proceedings, Protocol, Randomized Controlled Trial, Research, Review, Systematic Review; Language: English) | 47 |
| S33 | S3 AND S32 | 69 |
| S32 | S4 OR S5 OR S6 OR S7 OR S8 OR S9 OR S10 OR S11 OR S12 OR S13 OR S14 OR S15 OR S16 OR S17 | 228 |
| S31 | S3 AND S17 | 0 |
| S30 | S3 AND S16 | 8 |
| S29 | S3 AND S15 | 0 |
| S28 | S3 AND S14 | 0 |
| S27 | S3 AND S13 | 0 |
| S26 | S3 AND S12 | 11 |
| S25 | S3 AND S11 | 0 |
| S24 | S3 AND S10 | 4 |
| S23 | S3 AND S9 | 0 |
| S22 | S3 AND S8 | 1 |
| S21 | S3 AND S7 | 36 |
| S20 | S3 AND S6 | 0 |
| S19 | S3 AND S5 | 0 |
| S18 | S3 AND S4 | 0 |
| S17 | TX ("Spine Fine" OR "Giorgio Lofrese") | 0 |
| S16 | TX ("Neuro Therapy" OR "Monster Hub" OR "combined wellness solutions") | 10 |
| S15 | TX ("iAccess Life" OR "Rate & Review Accessibility" OR "iAccess Innovations") | 0 |
| S14 | TX (AccessiRep OR "Automatic Repetition Counter" OR "Cordical LC") | 0 |
| S13 | TX ("PVA ePubs" AND "Paralyzed Veterans of America") | 0 |
| S12 | TX (Injectful OR "Ehren Nelson" OR "Nicholas Barnes") | 11 |
| S11 | TX ("Dietitian's Tools" OR "Kinnereth LLC App Dev" OR "Grace Nutrition Consulting") | 0 |
| S10 | TX ("Maslow For People" OR "Maslow Pty") | 4 |
| S9 | TX ("JIB CALLS" OR "JIB Smart Home") | 0 |
| S8 | TX ("SNS Digital" AND "Paralyzed Veterans of America") | 1 |
| S7 | TX ("Paraplegia News" AND "Paralyzed Veterans of America") | 193 |
| S6 | TX ("Action Blocks" AND google) | 0 |
| S5 | TX ("Accessible Pakistan" OR SoftMining) | 0 |
| S4 | TX (Pilates AND DSN) | 0 |
| S3 | S1 OR S2 | 2,545,700 |
| S2 | TX (self-management OR self-care OR self-regulation OR self-monitoring) | 153,408 |
| S1 | TX (app* OR "mobile device" OR application* OR smartphone* OR application* OR iOS OR android OR tablet* OR "wireless technology" OR "wireless technologies" OR mhealth) | 2,490,894 |
|  |  |  |
| **Library, Information Science & Technology Abstracts** | | |
| **#** | **Query** | **Results** |
| S33 | S3 AND S32 | 0 |
| S32 | S4 OR S5 OR S6 OR S7 OR S8 OR S9 OR S10 OR S11 OR S12 OR S13 OR S14 OR S15 OR S16 OR S17 | 0 |
| S31 | S3 AND S17 | 0 |
| S30 | S3 AND S16 | 0 |
| S29 | S3 AND S15 | 0 |
| S28 | S3 AND S14 | 0 |
| S27 | S3 AND S13 | 0 |
| S26 | S3 AND S12 | 0 |
| S25 | S3 AND S11 | 0 |
| S24 | S3 AND S10 | 0 |
| S23 | S3 AND S9 | 0 |
| S22 | S3 AND S8 | 0 |
| S21 | S3 AND S7 | 0 |
| S20 | S3 AND S6 | 0 |
| S19 | S3 AND S5 | 0 |
| S18 | S3 AND S4 | 0 |
| S17 | TX ("Spine Fine" OR "Giorgio Lofrese") | 2 |
| S16 | TX ("Neuro Therapy" OR "Monster Hub" OR "combined wellness solutions") | 33 |
| S15 | TX ("iAccess Life" OR "Rate & Review Accessibility" OR "iAccess Innovations") | 0 |
| S14 | TX (AccessiRep OR "Automatic Repetition Counter" OR "Cordical LC") | 16 |
| S13 | TX ("PVA ePubs" AND "Paralyzed Veterans of America") | 1 |
| S12 | TX (Injectful OR "Ehren Nelson" OR "Nicholas Barnes") | 0 |
| S11 | TX ("Dietitian's Tools" OR "Kinnereth LLC App Dev" OR "Grace Nutrition Consulting") | 2 |
| S10 | TX ("Maslow For People" OR "Maslow Pty") | 6 |
| S9 | TX ("JIB CALLS" OR "JIB Smart Home") | 1 |
| S8 | TX ("SNS Digital" AND "Paralyzed Veterans of America") | 6 |
| S7 | TX ("Paraplegia News" AND "Paralyzed Veterans of America") | 2 |
| S6 | TX ("Action Blocks" AND google) | 91 |
| S5 | TX ("Accessible Pakistan" OR SoftMining) | 30 |
| S4 | TX (Pilates AND DSN) | 0 |
| S3 | S1 OR S2 | 203,534 |
| S2 | TX (self-management OR self-care OR self-regulation OR self-monitoring) | 1,820 |
| S1 | TX (app* OR "mobile device" OR application* OR smartphone* OR application* OR iOS OR android OR tablet* OR "wireless technology" OR "wireless technologies" OR mhealth) | 202,708 |
|  |  |  |
| **MEDLINE** | | |
| **#** | **Query** | **Results** |
| S34 | S3 AND S33 (Publication Type: Adaptive Clinical Trial, Case Reports, Case Study, Classical Article, Clinical Conference, Clinical Study, Clinical Trial, Clinical Trial Protocol, Clinical Trial, Phase I, Clinical Trial, Phase II, Clinical Trial, Phase III, Clinical Trial, Phase IV, Comparative Study, Conference, Congress, Controlled Clinical Trial, Equivalence Trial, Evaluation Study, Introductory Journal Article, Journal Article, Meta-Analysis, Observational Study, Pragmatic Clinical Trial, Randomized Controlled Trial, Report, Research, Review, Systematic Review, Technical Report, Twin Study, Validation Study; Language: English) | 4 |
| S33 | S3 AND S32 | 4 |
| S32 | S4 OR S5 OR S6 OR S7 OR S8 OR S9 OR S10 OR S11 OR S12 OR S13 OR S14 OR S15 OR S16 OR S17 | 12 |
| S31 | S3 AND S17 | 0 |
| S30 | S3 AND S16 | 4 |
| S29 | S3 AND S15 | 0 |
| S28 | S3 AND S14 | 0 |
| S27 | S3 AND S13 | 0 |
| S26 | S3 AND S12 | 0 |
| S25 | S3 AND S11 | 0 |
| S24 | S3 AND S10 | 0 |
| S23 | S3 AND S9 | 0 |
| S22 | S3 AND S8 | 0 |
| S21 | S3 AND S7 | 0 |
| S20 | S3 AND S6 | 0 |
| S19 | S3 AND S5 | 0 |
| S18 | S3 AND S4 | 0 |
| S17 | TX ("Spine Fine" OR "Giorgio Lofrese") | 3 |
| S16 | TX ("Neuro Therapy" OR "Monster Hub" OR "combined wellness solutions") | 7 |
| S15 | TX ("iAccess Life" OR "Rate & Review Accessibility" OR "iAccess Innovations") | 0 |
| S14 | TX (AccessiRep OR "Automatic Repetition Counter" OR "Cordical LC") | 0 |
| S13 | TX ("PVA ePubs" AND "Paralyzed Veterans of America") | 4 |
| S12 | TX (Injectful OR "Ehren Nelson" OR "Nicholas Barnes") | 1 |
| S11 | TX ("Dietitian's Tools" OR "Kinnereth LLC App Dev" OR "Grace Nutrition Consulting") | 0 |
| S10 | TX ("Maslow For People" OR "Maslow Pty") | 1 |
| S9 | TX ("JIB CALLS" OR "JIB Smart Home") | 5 |
| S8 | TX ("SNS Digital" AND "Paralyzed Veterans of America") | 259 |
| S7 | TX ("Paraplegia News" AND "Paralyzed Veterans of America") | 568 |
| S6 | TX ("Action Blocks" AND google) | 1,930 |
| S5 | TX ("Accessible Pakistan" OR SoftMining) | 1,466 |
| S4 | TX (Pilates AND DSN) | 10 |
| S3 | S1 OR S2 | 9,311,372 |
| S2 | TX (self-management OR self-care OR self-regulation OR self-monitoring) | 98,128 |
| S1 | TX (app* OR "mobile device" OR application* OR smartphone* OR application* OR iOS OR android OR tablet* OR "wireless technology" OR "wireless technologies" OR mhealth) | 9,257,407 |
|  |  |  |
| **PsycInfo** | | |
| **#** | **Query** | **Results** |
| 33 | 3 AND 32 | 3 |
| 32 | 4 OR 5 OR 6 OR 7 OR 8 OR 9 OR 10 OR 11 OR 12 OR 13 OR 14 OR 15 OR 16 OR 17 | 9 |
| 31 | 3 AND 17 | 3 |
| 30 | 3 AND 16 | 0 |
| 29 | 3 AND 15 | 0 |
| 28 | 3 AND 14 | 0 |
| 27 | 3 AND 13 | 0 |
| 26 | 3 AND 12 | 0 |
| 25 | 3 AND 11 | 0 |
| 24 | 3 AND 10 | 0 |
| 23 | 3 AND 9 | 0 |
| 22 | 3 AND 8 | 0 |
| 21 | 3 AND 7 | 0 |
| 20 | 3 AND 6 | 0 |
| 19 | 3 AND 5 | 0 |
| 18 | 3 AND 4 | 0 |
| 17 | "Neuro Therapy" OR "Monster Hub" OR "combined wellness solutions" | 7 |
| 16 | "Spine Fine" OR "Giorgio Lofrese" | 0 |
| 15 | "iAccess Life" OR "Rate & Review Accessibility" OR "iAccess Innovations" | 0 |
| 14 | AccessiRep OR "Automatic Repetition Counter" OR "Cordical LC" | 0 |
| 13 | "PVA ePubs" AND "Paralyzed Veterans of America" | 0 |
| 12 | Injectful OR "Ehren Nelson" OR "Nicholas Barnes" | 0 |
| 11 | "Dietitian's Tools" OR "Kinnereth LLC App Dev" OR "Grace Nutrition Consulting" | 0 |
| 10 | "Maslow For People" OR "Maslow Pty" | 0 |
| 9 | "JIB CALLS" OR "JIB Smart Home" | 0 |
| 8 | "SNS Digital" AND "Paralyzed Veterans of America" | 0 |
| 7 | "Paraplegia News" AND "Paralyzed Veterans of America" | 0 |
| 6 | "Action Blocks" AND google | 0 |
| 5 | "Accessible Pakistan" OR SoftMining | 0 |
| 4 | Pilates AND DSN | 0 |
| 3 | 1 OR 2 | 1,400,726 |
| 2 | self-management OR self-care OR self-regulation OR self-monitoring | 1,361,039 |
| 1 | app* OR "mobile device" OR application* OR smartphone* OR application* OR iOS OR android OR tablet* OR "wireless technology" OR "wireless technologies" OR mhealth | 59,707 |
|  |  |  |
| **Scopus** | | |
| **#** | **Query** | **Results** |
| 34 | (#3 AND #34) AND (LA==("ENGLISH")) | 101 |
| 33 | #3 AND #32 | 113 |
| 32 | #4 OR #5 OR #6 OR #7 OR #8 OR #9 OR #10 OR #11 OR #12 OR #13 OR #14 OR #15 OR #16 OR #17 | 131 |
| 31 | #3 AND #17 | 11 |
| 30 | #3 AND #16 | 34 |
| 29 | #3 AND #15 | 1 |
| 28 | #3 AND #14 | 0 |
| 27 | #3 AND #13 | 0 |
| 26 | #3 AND #12 | 2 |
| 25 | #3 AND #11 | 1 |
| 24 | #3 AND #10 | 0 |
| 23 | #3 AND #9 | 2 |
| 22 | #3 AND #8 | 0 |
| 21 | #3 AND #7 | 1 |
| 20 | #3 AND #6 | 5 |
| 19 | #3 AND #5 | 5 |
| 18 | #3 AND #4 | 3 |
| 17 | ALL("Spine Fine" OR "Giorgio Lofrese") | 14 |
| 16 | ALL("Neuro Therapy" OR "Monster Hub" OR "combined wellness solutions") | 45 |
| 15 | ALL("iAccess Life" OR "Rate & Review Accessibility" OR "iAccess Innovations") | 1 |
| 14 | ALL(AccessiRep OR "Automatic Repetition Counter" OR "Cordical LC") | 0 |
| 13 | ALL("PVA ePubs" AND "Paralyzed Veterans of America") | 0 |
| 12 | ALL(Injectful OR "Ehren Nelson" OR "Nicholas Barnes") | 2 |
| 11 | ALL("Dietitian's Tools" OR "Kinnereth LLC App Dev" OR "Grace Nutrition Consulting") | 1 |
| 10 | ALL("Maslow For People" OR "Maslow Pty") | 0 |
| 9 | ALL("JIB CALLS" OR "JIB Smart Home") | 2 |
| 8 | ALL("SNS Digital" AND "Paralyzed Veterans of America") | 0 |
| 7 | ALL("Paraplegia News" AND "Paralyzed Veterans of America") | 1 |
| 6 | ALL("Action Blocks" AND google) | 3 |
| 5 | ALL("Accessible Pakistan" OR SoftMining) | 5 |
| 4 | ALL(Pilates AND DSN) | 3 |
| 3 | S1 OR S2 | 49,447,908 |
| 2 | ALL(self-management OR self-care OR self-regulation OR self-monitoring) | 489,478 |
| 1 | ALL(app* OR "mobile device" OR application* OR smartphone* OR application* OR iOS OR android OR tablet* OR "wireless technology" OR "wireless technologies" OR mhealth) | 49,360,603 |
|  |  |  |
| **Web of Science** | | |
| **#** | **Query** | **Results** |
| S34 | (#3 AND #33) AND (LA==("ENGLISH") AND DT==("ARTICLE" OR "REVIEW")) | 4 |
| S33 | #3 AND #32 | 4 |
| S32 | #4 OR #5 OR #6 OR #7 OR #8 OR #9 OR #10 OR #11 OR #12 OR #13 OR #14 OR #15 OR #16 OR #17 | 13 |
| S31 | #3 AND #17 | 0 |
| S30 | #3 AND #16 | 2 |
| S29 | #3 AND #15 | 0 |
| S28 | #3 AND #14 | 0 |
| S27 | #3 AND #13 | 0 |
| S26 | #3 AND #12 | 1 |
| S25 | #3 AND #11 | 0 |
| S24 | #3 AND #10 | 0 |
| S23 | #3 AND #9 | 0 |
| S22 | #3 AND #8 | 0 |
| S21 | #3 AND #7 | 0 |
| S20 | #3 AND #6 | 0 |
| S19 | #3 AND #5 | 0 |
| S18 | #3 AND #4 | 0 |
| S17 | ALL=("Spine Fine" OR "Giorgio Lofrese") | 4 |
| S16 | ALL=("Neuro Therapy" OR "Monster Hub" OR "combined wellness solutions") | 5 |
| S15 | ALL=("iAccess Life" OR "Rate & Review Accessibility" OR "iAccess Innovations") | 0 |
| S14 | ALL=(AccessiRep OR "Automatic Repetition Counter" OR "Cordical LC") | 0 |
| S13 | ALL=("PVA ePubs" AND "Paralyzed Veterans of America") | 0 |
| S12 | ALL=(Injectful OR "Ehren Nelson" OR "Nicholas Barnes") | 2 |
| S11 | ALL=("Dietitian's Tools" OR "Kinnereth LLC App Dev" OR "Grace Nutrition Consulting") | 0 |
| S10 | ALL=("Maslow For People" OR "Maslow Pty") | 0 |
| S9 | ALL=("JIB CALLS" OR "JIB Smart Home") | 0 |
| S8 | ALL=("SNS Digital" AND "Paralyzed Veterans of America") | 0 |
| S7 | ALL=("Paraplegia News" AND "Paralyzed Veterans of America") | 0 |
| S6 | ALL=("Action Blocks" AND google) | 0 |
| S5 | ALL=("Accessible Pakistan" OR SoftMining) | 0 |
| S4 | ALL=(Pilates AND DSN) | 0 |
| S3 | #1 OR #2 | 17,547,770 |
| S2 | ALL=(self-management OR self-care OR self-regulation OR self-monitoring) | 95,372 |
| S1 | ALL=(app* OR "mobile device" OR application* OR smartphone* OR application* OR iOS OR android OR tablet* OR "wireless technology" OR "wireless technologies" OR mhealth) | 17,491,299 |
|  |  |  |
| **IEEE Xplore Digital Library** | | |
| **#** | **Query** | **Results** |
| 32 | #3 AND #17 | 0 |
| 31 | #3 AND #16 | 4 |
| 30 | #3 AND #15 | 0 |
| 29 | #3 AND #14 | 0 |
| 28 | #3 AND #13 | 0 |
| 27 | #3 AND #12 | 0 |
| 26 | #3 AND #11 | 0 |
| 25 | #3 AND #10 | 0 |
| 24 | #3 AND #9 | 0 |
| 23 | #3 AND #8 | 0 |
| 22 | #3 AND #7 | 0 |
| 21 | #3 AND #6 | 12 |
| 20 | #3 AND #5 (Filters: Conferences, Journals, Books, Early Access Articles) | 131 |
| 19 | #3 AND #5 | 132 |
| 18 | #3 AND #4 | 0 |
| 17 | ("Full Text Only":"Spine Fine" OR "Full Text Only":"Giorgio Lofrese") | 0 |
| 16 | ("Full Text Only":"Neuro Therapy" OR "Full Text Only":"Monster Hub" OR "Full Text Only":"combined wellness solutions") | 4 |
| 15 | ("Full Text Only":"iAccess Life" OR "Full Text Only":"Rate & Review Accessibility" OR "Full Text Only":"iAccess Innovations") | 0 |
| 14 | ("Full Text Only":AccessiRep OR "Full Text Only":"Automatic Repetition Counter" OR "Full Text Only":"Cordical LC") | 0 |
| 13 | ("Full Text Only":"PVA ePubs" AND "Full Text Only":"Paralyzed Veterans of America") | 0 |
| 12 | ("Full Text Only":Injectful OR "Full Text Only":"Ehren Nelson" OR "Full Text Only":"Nicholas Barnes") | 0 |
| 11 | ("Full Text Only":"Dietitian's Tools" OR "Full Text Only":"Kinnereth LLC App Dev" OR "Full Text Only":"Grace Nutrition Consulting") | 0 |
| 10 | ("Full Text Only":"Maslow FOR "Full Text Only":People" OR "Full Text Only":"Maslow Pty") | 0 |
| 9 | ("Full Text Only":"JIB CALLS" OR "Full Text Only":"JIB Smart Home") | 0 |
| 8 | ("Full Text Only":"SNS Digital" AND "Full Text Only":"Paralyzed Veterans of America") | 0 |
| 7 | ("Full Text Only":"Paraplegia News" AND "Full Text Only":"Paralyzed Veterans of America") | 0 |
| 6 | ("Full Text Only":"Action Blocks" AND "Full Text Only":google) | 12 |
| 5 | ("Full Text Only":"Accessible Pakistan" OR "Full Text Only":SoftMining) | 132 |
| 4 | ("Full Text Only":Pilates AND "Full Text Only":DSN) | 0 |
| 3 | #1 OR #2 | 20864 |
| 2 | ("Full Text Only":self-management OR "Full Text Only":self-care OR "Full Text Only":self-regulation OR "Full Text Only":self-monitoring) | 21117 |
| 1 | ("Full Text Only":app* OR "Full Text Only":"mobile device" OR "Full Text Only":application* OR "Full Text Only":smartphone* OR "Full Text Only":application* OR "Full Text Only":iOS OR "Full Text Only":android OR "Full Text Only":tablet* OR "Full Text Only":"wireless technology" OR "Full Text Only":"wireless technologies" OR "Full Text Only":mhealth) | 4965245 |
|  |  |  |
| **ACM Digital Library** | | |
| **#** | **Query** |  |
| 33 | #3 AND #32 | 150 |
| 32 | #4 OR #5 OR #6 OR #7 OR #8 OR #9 OR #10 OR #11 OR #12 OR #13 OR #14 OR #15 OR #16 OR #17 | 150 |
| 31 | #3 AND #17 | 1 |
| 30 | #3 AND #16 | 0 |
| 29 | #3 AND #15 | 0 |
| 28 | #3 AND #14 | 0 |
| 27 | #3 AND #13 | 0 |
| 26 | #3 AND #12 | 0 |
| 25 | #3 AND #11 | 0 |
| 24 | #3 AND #10 | 0 |
| 23 | #3 AND #9 | 0 |
| 22 | #3 AND #8 | 0 |
| 21 | #3 AND #7 | 0 |
| 20 | #3 AND #6 | 17 |
| 19 | #3 AND #5 | 3 |
| 18 | #3 AND #4 | 0 |
| 17 | [All:"Spine Fine"] OR [All:"Giorgio Lofrese"] | 1 |
| 16 | [All:"Neuro Therapy"] OR [All:"Monster Hub"] OR [All:"combined wellness solutions"] | 0 |
| 15 | [All:"iAccess Life"] OR [All:"Rate & Review Accessibility"] OR [All:"iAccess Innovations"] | 0 |
| 14 | [All:AccessiRep] OR [All:"Automatic Repetition Counter"] OR [All:"Cordical LC"] | 0 |
| 13 | [All:"PVA ePubs"] AND [All:"Paralyzed Veterans of America"] | 0 |
| 12 | [All:Injectful] OR [All:"Ehren Nelson"] OR [All:"Nicholas Barnes"] | 0 |
| 11 | [All:"Dietitian's Tools"] OR [All:"Kinnereth LLC App Dev"] OR [All:"Grace Nutrition Consulting"] | 0 |
| 10 | [All:"Maslow FOR All:People"] OR [All:"Maslow Pty"] | 0 |
| 9 | [All:"JIB CALLS"] OR [All:"JIB Smart Home"] | 0 |
| 8 | [All:"SNS Digital"] AND [All:"Paralyzed Veterans of America"] | 0 |
| 7 | [All:"Paraplegia News"] AND [All:"Paralyzed Veterans of America"] | 0 |
| 6 | [All:"Action Blocks"] AND [All:google] | 17 |
| 5 | [All:"Accessible Pakistan"] OR [All:SoftMining] | 3 |
| 4 | [All:Pilates] AND [All:DSN] | 0 |
| 3 | #1 OR #2 | 640045 |
| 2 | [All:self-management] OR [All:self-care] OR [All:self-regulation] OR [All:self-monitoring] | 445886 |
| 1 | [All:app*] OR [All:"mobile device"] OR [All:application*] OR [All:smartphone*] OR [All:application*] OR [All:iOS] OR [All:android] OR [All:tablet*] OR [All:"wireless technology"] OR [All:"wireless technologies"] OR [All:mhealth] | 630,643 |
|  |  |  |
| **EMBASE** | | |
| **#** | **Query** | **Results** |
| #34 | (#3 AND #33) AND ([article]/lim OR [conference paper]/lim OR [review]/lim OR [preprint]/lim) AND [english]/lim AND ([embase]/lim OR [embase classic]/lim) | 14 |
| #33 | #3 AND #32 | 24 |
| #32 | #4 OR #5 OR #6 OR #7 OR #8 OR #9 OR #10 OR #11 OR #12 OR #13 OR #14 OR #15 OR #16 OR #17 OR #18 OR #19 OR #20 OR #21 OR #22 OR #23 | 46 |
| #31 | #3 AND #17 | 14 |
| #30 | #3 AND #16 | 7 |
| #29 | #3 AND #15 | 0 |
| #28 | #3 AND #14 | 0 |
| #27 | #3 AND #13 | 0 |
| #26 | #3 AND #12 | 2 |
| #25 | #3 AND #11 | 0 |
| #24 | #3 AND #10 | 0 |
| #23 | #3 AND #9 | 0 |
| #22 | #3 AND #8 | 0 |
| #21 | #3 AND #7 | 0 |
| #20 | #3 AND #6 | 0 |
| #19 | #3 AND #5 | 0 |
| #18 | #3 AND #4 | 0 |
| #17 | "Neuro Therapy" OR "Monster Hub" OR "combined wellness solutions" | 23 |
| #16 | "Spine Fine" OR "Giorgio Lofrese" | 18 |
| #15 | "iAccess Life" OR "Rate & Review Accessibility" OR "iAccess Innovations" | 0 |
| #14 | AccessiRep OR "Automatic Repetition Counter" OR "Cordical LC" | 0 |
| #13 | "PVA ePubs" AND "Paralyzed Veterans of America" | 0 |
| #12 | Injectful OR "Ehren Nelson" OR "Nicholas Barnes" | 4 |
| #11 | "Dietitian* Tools" OR "Kinnereth LLC App Dev" OR "Grace Nutrition Consulting" | 0 |
| #10 | "Maslow For People" OR "Maslow Pty" | 0 |
| #9 | "JIB CALLS" OR "JIB Smart Home" | 0 |
| #8 | "SNS Digital" AND "Paralyzed Veterans of America" | 0 |
| #7 | "Paraplegia News" AND "Paralyzed Veterans of America" | 0 |
| #6 | "Action Blocks" AND google | 0 |
| #5 | "Accessible Pakistan" OR SoftMining | 0 |
| #4 | Pilates AND DSN | 0 |
| #3 | #1 OR #2 | 11024118 |
| #2 | self-management OR self-care OR self-regulation OR self-monitoring | 116283 |
| #1 | app* OR "mobile device" OR application* OR smartphone* OR application* OR iOS OR android OR tablet* OR "wireless technology" OR "wireless technologies" OR mhealth | 10954253 |
