# Supplementary material for: Self-Management Support Apps for Spinal Cord Injury: Results of a Systematic Search in App Stores and Mobile App Rating Scale Evaluation
Source: JMIR Mhealth Uhealth. 2024 Dec 19;12:e53677. doi: 10.2196/53677 (PMC11695972; doi:10.2196/53677)
Supplement: Multimedia Appendix 3 [file mhealth_v12i1e53677_app3.docx]

Multimedia Appendix 3: Characteristics of the 13 identified self-management apps for spinal cord injury (SCI).

| App name (launch year) citation, developer name, country, and target age | Description | Self-management focus areas | App store category and emergent category | Supported devices, latest app version, last updated year, file size, and earliest operating system version supported | Country availability | Supported languages | Cost (US $) and in-app purchases |
| --- | --- | --- | --- | --- | --- | --- | --- |
| Pilates (2013) [47], DSN Inc, Belarus | It consists of a series of Pilates exercises and respiratory techniques aiming to develop mobility and flexibility, tighten and strengthen muscles, help restore muscle balance, improve endurance and tone, and reduce stress. | Physical activity promotion | Health and fitness (physical or mental exercises) | Smartphone, 2.5.9 (2019), 53.48 MB, Android 4 | Algeria, Argentina, Armenia, Australia, Austria, Belarus, Belgium, Brazil, Bulgaria, Canada, Chile, China, Colombia, Croatia, Cyprus, Czech Republic, Denmark, Egypt, Estonia, Finland, France, Georgia, Germany, Greece, Hong Kong, Hungary, India, Indonesia, Iran, Ireland, Israel, Italy, Japan, Jordan, Korea, Latvia, Lebanon, Lithuania, Luxembourg, Malaysia, Mexico, Morocco, Netherlands, New Zealand, Nigeria, Norway, Pakistan, Peru, Philippines, Poland, Portugal, Romania, Russia, Saudi Arabia, Serbia, Singapore, Slovakia, Slovenia, South Africa, Spain, Sweden, Switzerland, Taiwan, Thailand, Tunisia, Ukraine, United Arab Emirates, the United Kingdom, the United States, and Vietnam | Czech, Danish, German, Greek, English, Spanish, French, Italian, Portuguese, Russian, Slovak, Swedish, and Turkish | 9.99, no |
| Action Blocks (2020) [35], Google LLC, the United States | It helps to perform a routine action on mobile phones or tablets easily by using customizable buttons on the screen. The app requires the use of Google Assistant. | Activities of daily living | Tools (accessibility tools) | Smartphone and tablet, 1.5.440171149 (2022), 10.02 MB, Android 6 | Algeria, Argentina, Armenia, Australia, Austria, Belarus, Belgium, Brazil, Bulgaria, Canada, Chile, China, Colombia, Croatia, Cyprus, Czech Republic, Denmark, Egypt, Estonia, Finland, France, Georgia, Germany, Greece, Hong Kong, Hungary, India, Indonesia, Iran, Ireland, Israel, Italy, Japan, Jordan, Korea, Latvia, Lebanon, Lithuania, Luxembourg, Malaysia, Mexico, Morocco, the Netherlands, New Zealand, Nigeria, Norway, Pakistan, Peru, Philippines, Poland, Portugal, Romania, Russia, Saudi Arabia, Serbia, Singapore, Slovakia, Slovenia, South Africa, Spain, Sweden, Switzerland, Taiwan, Thailand, Tunisia, Ukraine, United Arab Emirates, the United Kingdom, the United States, and Vietnam | German, English, Spanish, French, Italian, Japanese, and Portuguese | Free, no |
| PN–Paraplegia News^a^ (2017) [44], Paralyzed Veterans of America, the United States | It is a digital version of the print publication from the Paralyzed Veterans of America organization. | Health literacy, activities of daily living | News and magazines (communication or information or knowledge) | Smartphone and tablet, 7.0.8 (2020), 20.97 MB, Android 4.4 | Algeria, Argentina, Armenia, Australia, Austria, Belarus, Belgium, Brazil, Bulgaria, Canada, Chile, Colombia, Croatia, Cyprus, Czech Republic, Denmark, Egypt, Estonia, Finland, France, Germany, Greece, Hong Kong, Hungary, India, Indonesia, Iran, Ireland, Israel, Italy, Japan, Jordan, Korea, Latvia, Lebanon, Lithuania, Luxembourg, Malaysia, Mexico, Morocco, the Netherlands, New Zealand, Nigeria, Norway, Pakistan, Peru, Philippines, Poland, Portugal, Romania, Russia, Saudi Arabia, Serbia, Singapore, Slovakia, Slovenia, South Africa, Spain, Sweden, Switzerland, Taiwan, Thailand, Tunisia, Turkey, Ukraine, United Arab Emirates, the United Kingdom, the United States, and Vietnam | English | 19.99, yes |
| SNS Digital^a^ (2017) [45], Paralyzed Veterans of America, the United States | It is a digital version of the print publication from the Paralyzed Veterans of America organization. The articles cover all topics related to wheelchair sports and recreation. | Physical activity promotion, health literacy, and activities of daily living | Sports (communication or information or knowledge) | Smartphone and tablet, 7.0.8 (2020), 20.97 MB, Android 4.4 | Algeria, Argentina, Armenia, Australia, Austria, Belarus, Belgium, Brazil, Bulgaria, Canada, Chile, Colombia, Croatia, Cyprus, Czech Republic, Denmark, Egypt, Estonia, Finland, France, Germany, Greece, Hong Kong, Hungary, India, Indonesia, Ireland, Israel, Italy, Japan, Jordan, Korea, Latvia, Lebanon, Lithuania, Luxembourg, Malaysia, Mexico, Morocco, the Netherlands, New Zealand, Nigeria, Norway, Pakistan, Peru, Philippines, Poland, Portugal, Romania, Russia, Saudi Arabia, Serbia, Singapore, Slovakia, Slovenia, South Africa, Spain, Sweden, Switzerland, Taiwan, Thailand, Tunisia, Turkey, Ukraine, United Arab Emirates, the United Kingdom, the United States, and Vietnam | English | 14.99, yes |
| JIB CALLS (2020) [36], JIB Smart Home, France | It allows to make a hands-free call with prior personal configuration, such as authorization of the phone numbers, available timeslots, tone rings, and others. The authorized calls are accepted and put on speakerphone. | Activities of daily living | Communication (communication or information or knowledge) | Smartphone, 2.0 (2022), 8.18 MB, Android 6 | Algeria, Australia, Belgium, Brazil, Canada, France, Germany, Luxembourg, Switzerland, Tunisia, the United Kingdom, and the United States | German, English, and French | 0.99, yes |
| Disability Care App^b^ (2020) [37], Maslow For People, Australia | A voice-enabled life assistant that helps to create and follow up personal rehabilitation routines, such as storing all exercises, care guides, and tips in one place; designing one’s own care routine and monitoring rehabilitation and care progress; communicating with health specialists; and accessing the best practices in health education. | Therapeutic exercise and activities of daily living | Health and fitness (daily routine assistant) | Smartphone and tablet, 2.0.15 (2022), 116.39 MB, Android 5.1 | Australia, Germany, New Zealand, and the United States | English | Free, no |
| Dietitian’s Tools (2019) [38], Kinnereth LLC App Dev, the United States | It is an aid for healthy diet calculations. The app offers various options for more precise diet calculations (such as kilocalories, proteins, and fluid intake) according to the needs of different health conditions. It targets patients with paraplegia and quadriplegia among other conditions. | Medicating and dieting | Health and fitness (daily routine assistant) | Smartphone, 1.0 (2019), 32.51 MB, Android 4.1 | Algeria, Argentina, Armenia, Australia, Austria, Belarus, Belgium, Brazil, Bulgaria, Canada, Chile, Colombia, Croatia, Cyprus, Czech Republic, Denmark, Egypt, Estonia, Finland, France, Georgia, Germany, Greece, Hong Kong, Hungary, India, Indonesia, Ireland, Israel, Italy, Japan, Jordan, Korea, Latvia, Lebanon, Lithuania, Luxembourg, Malaysia, Mexico, Morocco, the Netherlands, New Zealand, Nigeria, Norway, Pakistan, Peru, Philippines, Poland, Portugal, Romania, Russia, Saudi Arabia, Serbia, Singapore, Slovakia, Slovenia, South Africa, Spain, Sweden, Switzerland, Taiwan, Thailand, Tunisia, Turkey, Ukraine, United Arab Emirates, the United Kingdom, the United States, and Vietnam | English and French | 1.99, no |
| Injectful^a^ (2020) [39], Injectful LLC, the United States | The app provides mindfulness techniques and education to help manage pain. Consultation with a health professional is recommended before using the app. It targets all patients undergoing injections and interventional pain procedures or people with some degree of chronic pain. | Pain management | Health and Fitness (physical or mental exercises) | Smartphone and tablet, 1.0 (2020), 16.78 MB, Android 4.4 | Algeria, Argentina, Armenia, Australia, Austria, Belarus, Belgium, Brazil, Bulgaria, Canada, Chile, Colombia, Croatia, Cyprus, Czech Republic, Denmark, Egypt, Estonia, Finland, France, Georgia, Germany, Greece, Hong Kong, Hungary, India, Indonesia, Ireland, Israel, Italy, Japan, Jordan, Korea, Latvia, Lebanon, Lithuania, Luxembourg, Malaysia, Mexico, Morocco, the Netherlands, New Zealand, Nigeria, Norway, Pakistan, Peru, Philippines, Poland, Portugal, Romania, Russia, Saudi Arabia, Serbia, Singapore, Slovakia, Slovenia, South Africa, Spain, Sweden, Switzerland, Taiwan, Thailand, Tunisia, Turkey, Ukraine, United Arab Emirates, the United Kingdom, the United States, and Vietnam | English | 9.99, no |
| PVA ePubs (2013) [46], Paralyzed Veterans of America, the United States | It is a digital version of the print publication from the Paralyzed Veterans of America organization including self-care guidelines (“Yes, you can”), clinical practice guidelines, and other self-help publications. It targets people with SCI. | Health literacy and activities of daily living | Medical (communication or information or knowledge) | Smartphone and tablet, 4.11.2 (2022), 42.47 MB, iOS 11 | Algeria, Argentina, Armenia, Australia, Austria, Belarus, Belgium, Brazil, Bulgaria, Canada, Chile, China, Colombia, Croatia, Cyprus, Czech Republic, Denmark, Dominican Republic, Egypt, Estonia, Finland, France, Georgia, Germany, Greece, Hong Kong, Hungary, India, Indonesia, Ireland, Israel, Italy, Japan, Jordan, Korea, Latvia, Lebanon, Lithuania, Luxembourg, Malaysia, Mexico, Morocco, the Netherlands, New Zealand, Nigeria, Norway, Pakistan, Peru, Philippines, Poland, Portugal, Romania, Russia, Saudi Arabia, Serbia, Singapore, Slovakia, Slovenia, South Africa, Spain, Sweden, Switzerland, Taiwan, Thailand, Tunisia, Turkey, Ukraine, United Arab Emirates, the United Kingdom, the United States, and Vietnam | English | Free, yes |
| AccessiRep (2019) [40], Cordilac LC, the United States | The app counts the exercises with the help of motion sensors that detect any activity. It targets people with SCI. | Physical activity promotion | Health and fitness (physical or mental exercises) | Smartphone and tablet, 1.1 (2020), 25.65 MB, iOS 9 | Algeria, Argentina, Armenia, Australia, Austria, Belarus, Belgium, Brazil, Bulgaria, Canada, Chile, China, Colombia, Croatia, Cyprus, Czech Republic, Denmark, Dominican Republic, Egypt, Estonia, Finland, France, Georgia, Germany, Greece, Hong Kong, Hungary, India, Indonesia, Ireland, Israel, Italy, Japan, Jordan, Korea, Latvia, Lebanon, Lithuania, Luxembourg, Malaysia, Mexico, Morocco, the Netherlands, New Zealand, Nigeria, Norway, Pakistan, Peru, Philippines, Poland, Portugal, Romania, Russia, Saudi Arabia, Serbia, Singapore, Slovakia, Slovenia, South Africa, Spain, Sweden, Switzerland, Taiwan, Thailand, Tunisia, Turkey, Ukraine, United Arab Emirates, the United Kingdom, the United States, and Vietnam | English | 1.99, no |
| iAccess Life– Accessibility^a^ (2019) [41], iAccess Innovations, the United States | It allows people with disabilities, wheelchair users, and users of mobility aids (canes and walkers) to share their experience with accessibility at various venues and events around the world. | Mobility | Lifestyle (mobility) | Smartphone, 2.2 (2020), 68.59 MB, iOS 12 | Algeria, Argentina, Armenia, Australia, Austria, Belarus, Belgium, Brazil, Bulgaria, Canada, Chile, China, Colombia, Croatia, Cyprus, Czech Republic, Denmark, Dominican Republic, Egypt, Estonia, Finland, France, Georgia, Germany, Greece, Hong Kong, Hungary, India, Indonesia, Ireland, Israel, Italy, Japan, Jordan, Korea, Latvia, Lebanon, Lithuania, Luxembourg, Malaysia, Mexico, Morocco, the Netherlands, New Zealand, Nigeria, Norway, Pakistan, Peru, Philippines, Poland, Portugal, Romania, Russia, Saudi Arabia, Serbia, Singapore, Slovakia, Slovenia, South Africa, Spain, Sweden, Switzerland, Taiwan, Thailand, Tunisia, Turkey, Ukraine, United Arab Emirates, the United Kingdom, the United States, and Vietnam | English | Free, no |
| Neuro Therapy (2019) [42], Monster Hub, Australia | It provides a tailored home exercise program. It targets people with SCI and considers comorbidities. | Therapeutic exercise and physical activity promotion | Health and fitness (physical or mental exercises) | Smartphone, 0.92.2 (2019), 82.71 MB, iOS 12 | Algeria, Argentina, Armenia, Australia, Austria, Belarus, Belgium, Brazil, Bulgaria, Canada, Chile, China, Colombia, Croatia, Cyprus, Czech Republic, Denmark, Dominican Republic, Egypt, Estonia, Finland, France, Georgia, Germany, Greece, Hong Kong, Hungary, India, Indonesia, Ireland, Israel, Italy, Japan, Jordan, Korea, Latvia, Lebanon, Lithuania, Luxembourg, Malaysia, Mexico, Morocco, the Netherlands, New Zealand, Nigeria, Norway, Pakistan, Peru, Philippines, Poland, Portugal, Romania, Russia, Saudi Arabia, Serbia, Singapore, Slovakia, Slovenia, South Africa, Spain, Sweden, Switzerland, Taiwan, Thailand, Tunisia, Turkey, Ukraine, United Arab Emirates, the United Kingdom, the United States, and Vietnam | English | Free, no |
| Spine Fine (2021) [43], Giorgio Lofrese, Italy | It helps to monitor the effectiveness of the surgery or rehabilitation path through the evaluation of common hand gestures. | Therapeutic exercise and physical activity promotion | Medical (physical or mental exercises) | Tablet, 1.4.3 (2022), 16.16 MB, iOS 14.5 | Algeria, Argentina, Armenia, Australia, Austria, Belarus, Belgium, Brazil, Bulgaria, Canada, Chile, China, Colombia, Croatia, Cyprus, Czech Republic, Denmark, Egypt, Estonia, Finland, France, Georgia, Germany, Greece, Hong Kong, Hungary, India, Indonesia, Ireland, Israel, Italy, Japan, Jordan, Korea, Latvia, Lebanon, Lithuania, Luxembourg, Malaysia, Mexico, Morocco, the Netherlands, New Zealand, Nigeria, Norway, Pakistan, Peru, Philippines, Poland, Portugal, Romania, Russia, Saudi Arabia, Serbia, Singapore, Slovakia, Slovenia, South Africa, Spain, Sweden, Switzerland, Taiwan, Thailand, Tunisia, Turkey, Ukraine, United Arab Emirates, the United Kingdom, the United States, and Vietnam | English and Italian | Free, no |

^a^App available in both app stores
